# Role of Advanced Glycation End Products and Mitohormesis in Cancer Development and Progression

**DOI:** 10.3390/antiox14101165

**Published:** 2025-09-25

**Authors:** Donghyun Kim, Kyung-Nam Choi, Jong-In Park, Eun-Hye Kim, Arshad Majid, Ok-Nam Bae

**Affiliations:** 1 College of Pharmacy, Institute of Pharmaceutical Science and Technology, Hanyang University, Ansan 15588, Republic of Korea; donghyun6@hanyang.ac.krchoikn0615@hanyang.ac.kr (K.-N.C.); whddls0804@hanyang.ac.kr (J.-I.P.); 2 College of Pharmacy, Keimyung University, Daegu 42601, Republic of Korea; 3 College of Pharmacy, Kyungsung University, Busan 48434, Republic of Korea; dldh615@ks.ac.kr; 4 Division of Neuroscience, School of Medicine and Population Health, The University of Sheffield, Sheffield S10 2TN, UK; arshad.majid@sheffield.ac.uk

**Keywords:** advanced glycation end products (AGEs), mitochondria, mitohormesis, cancer, tumorigenesis

## Abstract

Advanced glycation end products (AGEs) are molecules formed via non-enzymatic reactions between reactive dicarbonyls and macromolecules, including proteins, lipids, or DNA. Mitochondria sense and integrate stress signals and induce changes in cellular function by regulating metabolism, redox balance, and proteostasis to maintain homeostasis, a process known as mitohormesis. Dysregulation of cellular metabolism and redox imbalance are the major driving forces behind the increased production of intracellular reactive dicarbonyls and AGEs. Although the association between increased reactive dicarbonyl levels and cancer development has been investigated, its causal relationship remains controversial. This review integrates recent evidence on the association between increased levels of reactive dicarbonyls and mitochondrial dysfunction and provides mechanistic insights into carcinogenesis associated with AGE-mediated disruption of mitohormesis.

## 1. Introduction

Glycation compounds are both endogenously and exogenously derived from the Maillard reaction between reducing sugars or reactive dicarbonyls and macromolecules, including proteins, lipids, and DNA. Depending on the stage of the Maillard reaction, glycation compounds are classified as Amadori compounds (early stage), dicarbonyl compounds (advanced stage), and advanced glycation end products (AGEs; final stage) [1]. 3-Desoxyglucosulose, 3-desoxypentosulose, glyoxal, and methylglyoxal (MG) are representative highly reactive dicarbonyl compounds and precursors of pyrraline, formyline, Nε-(carboxylmethyl)-l-lysine (CML), and Nδ-(5-hydro-5-methyl-4-imidazolon-2-yl)-l-ornithine (MG-H_1_), respectively [1].

Emerging issues in the human health risk assessment of AGEs include evaluation of the additional body burden resulting from dietary consumption in relation to its basal endogenous production [2]. Reactive dicarbonyl compounds are endogenously produced as a byproduct of glycolysis in the cytoplasm, and their levels are regulated by the intracellular detoxification mechanism of glyoxalase [1]. Accumulating evidence has shown that AGEs are associated with various chronic diseases, and endogenous AGE formation has attracted attention as an important pathological mechanism of carcinogenesis [3,4].

Cancer is defined by the National Cancer Institute as a disease in which some of the body’s cells grow uncontrollably and spread to other parts of the body [5]. Hallmarks of cancer proposed by Hanahan and Weinberg include the dysregulation of cellular metabolism, sustaining proliferative signaling, evading growth suppressors, avoiding immune destruction, enabling replicative immortality, tumor-promoting inflammation, activating invasion and metastasis, inducing or accessing the vasculature, genome instability and mutation, and resistance to cell death [6]. These processes are associated with remarkable changes in mitochondrial function [7]. Alterations in metabolite and reactive oxygen species (ROS) production [8], as well as activation of stress responses [9,10], are considered key molecular events linked to mitohormesis and tumorigenesis.

Hormesis is an adaptive strategy with a biphasic dose–response relationship, characterized by low-dose stimulation and high-dose inhibition [11]. A stimulatory response that is typically 30–60% above control levels is a characteristic feature of the hormetic dose–response [11]. Low doses of chemical stress can induce a hormetic response, positively regulating biological functions to restore homeostasis [12]. Hormesis is important not only for the survival of normal cells but also in tumorigenesis [13,14]. Formation of stress granules (SGs) and production of mitochondrial ROS have been proposed as representative hormetic adaptive responses in tumors [13].

Mitohormesis is a process that senses and integrates stress signals and induces changes in cellular function by regulating metabolism, redox balance, and proteostasis to increase mitochondrial stress resistance [15]. Mitochondrial stress signaling is mediated by mitochondrial membrane potential, production of ROS, mitochondrial NAD^+^, tricarboxylic acid (TCA) cycle intermediates, and mitochondria-derived peptides [15,16]. Mitochondrial stress can activate the integrated stress response (ISR) or mitochondrial unfolded protein response (UPR^mt^), leading to the translation of nuclear-encoded hormetic response genes [15]. Under physiological conditions, mitohormesis is associated with longevity, yet it is also implicated in carcinogenesis and metastasis [13]. Mitochondria play an essential role in tumor growth, and activation of the UPR^mt^ prevents tumor cell death by decreasing mitochondrial damage caused by proteotoxic stress [17].

Accumulating evidence indicates that the regulation of endogenous reactive dicar-bonyls is associated with hormesis [18], and increased AGEs act as a mitochondrial stressor, representing an important underlying mechanism of various age-related diseases [19,20]. In this review, we aim to present the carcinogenic mechanisms of AGEs by synthesizing the latest knowledge on the role of mitochondria in AGEs-induced carcinogenesis.

## 2. Role of Mitochondria in Tumorigenesis and Cancer Progression

### 2.1. Mitochondrial Oxidative Stress in Cancer

Mitochondria are multifunctional organelles that regulate energy production, redox balance, and proteostasis in cells, with over 20 cellular functions [21]. Mitochondrial ROS production, alterations in mitochondrial DNA (mtDNA), dysregulation of mitochondrial dynamics, and mitochondrial retrograde signaling have been suggested to be the major initiating events associated with tumorigenesis and cancer progression [22].

Various mechanisms of tumorigenesis are associated with ROS production. In cells, there are multiple sources of ROS generation, including nicotinamide adenine dinucleotide phosphate oxidase (NOX), xanthine oxidase (XO), cytochrome P450, and the mitochondrial electron transport chain (ETC) [23]. Among them, mitochondria are major source of ROS, and their redox regulation is associated with proliferation and resistance to cancer cell death [22]. Overproduction of ROS can be detrimental to cell survival, as it increases DNA damage and genomic instability. In addition, acting as a secondary messenger, ROS contributes to the activation of mitogenic signaling pathways, including PI3K/AKT/mTOR and mitogen-activated protein kinase (MAPK)/extracellular signal-regulated kinase (ERK) [24]. Moreover, thiol-disulfide transformation upon ROS generation leads to inactivation of PTEN and neoplastic progression [24]. METTL17, a mitochondrial protein, increases the resistance to ferroptosis of colorectal cancer cells by suppressing mitochondrial lipid peroxidation and ROS [25].

Mitochondrial ROS production by cancer cells and immunosuppressive immune cells is associated with immune tolerance to tumors [26]. Recently, Martins et al. [27] reported that programmed cell death protein 1 (PD-1) promotes Merkel cell carcinoma, an aggressive skin cancer, by activating mTOR/mitochondrial ROS. Lon is a mitochondrial protease with both proteolytic and chaperone activities, playing a key role in mitochondrial protein quality control [26], and it has been shown to induce mitochondrial ROS production through interactions with mitochondrial matrix enzymes. Kuo et al. [28] reported that Lon-induced mitochondrial ROS stress promotes cancer development through the formation of an immunosuppressive tumor microenvironment.

The role of the mitochondrial antioxidant defense mechanism in carcinogenesis has been investigated [29,30]. Sirtuin 3 (SIRT3) is an NAD^+^-dependent deacetylase localized in both the mitochondria and nucleus. It inhibits mitochondrial ROS production by activating antioxidant enzymes such as superoxide dismutase 2 (SOD2) and catalase [31]. The role of SIRT3 in cancer development has been reported to include both tumor-suppressive [32] and tumor-promoting functions. SIRT3 can suppress tumor growth by downregulating hypoxia-inducible factor 1α (HIF-1α) [33]. Additionally, deacetylation of SOD2 has been linked to cancer stem cell formation via activation of HIF-2α, contributing to tumor aggressiveness and poor clinical outcomes [34,35]. In contrast, other studies have reported that upregulated SIRT3 expression in chronic lymphocytic leukemia (CLL) cells is associated with tumor cell survival by facilitating the elimination of superoxide anions [36].

### 2.2. Alteration of mtDNA in Cancer

Altered mtDNA copy number and mutation are critical events associated with the alteration of mitochondrial function and cellular fate [37]. The association between mtDNA copy number and the development and metastasis of various cancer types has been investigated. In a prospective cohort study, increased mtDNA copy number was positively associated with the risk of lung cancer [38]. Increased mtDNA copy number in peripheral blood cells has been associated with an increased risk of breast cancer [39]. Another study reported that the blood mtDNA copy number was positively associated with the risk of breast cancer and mediated the relationship between the environmental toxicants perfluoroalkyl substances and breast cancer [40]. However, in a cohort of primary breast tumor specimens from patients, low mtDNA content was associated with poor prognosis of ten-year distant metastasis-free survival [41]. In an animal study, increased mtDNA content was associated with increased tumor size, and mtDNA depletion prevented the survival and metastasis of tumors in a xenograft mouse model of colorectal cancer [42].

Mutations in mtDNA are associated with various cancer risks. In a cohort study, mutation in plasma cell-free mtDNA (mt.16093T > C) was associated with an increased risk of breast cancer [43]. In addition, mtDNA-mutated breast cancer cells showed increased metastatic potential in a mouse model [44]. In a xenograft model of prostate cancer, mtDNA mutations (mt.8993T > G or mt.6124T > C) significantly promoted tumor growth compared with wild-type cybrids [45,46,47]. Another study reported that a mitochondrially encoded cytochrome B (CYTB) gene mutation (a 21-bp deletion) promoted the growth and bioenergetic capacity of bladder cancer-derived cells [48].

Mutations in mtDNA can affect the tumor microenvironment. Recent studies have reported mitochondrial transfer of cancer cells to tumor-infiltrating lymphocytes (TILs), and TIL-acquired mtDNA mutations from cancer cells showed metabolic abnormalities and senescence [49]. In addition, mtDNA mutation in mt-ND5 (mt.12,436G>A or mt.11,944G>A) promoted a Warburg-like metabolic shift in melanoma [50]. In addition, mtDNA mutation affected therapeutic response to immune checkpoint inhibitors. An mtDNA mutation in tumor tissue is a poor prognostic factor for immune checkpoint inhibitors in patients with melanoma or NSCLC [49]. In an animal study, mtDNA mutation in mt-ND5 increased response to checkpoint blockade of anti-PD1 in a mouse tumor xenograft model [50].

### 2.3. Alteration of Mitochondrial Dynamics in Cancer

Mitochondrial dynamics encompass the processes of changes in the morphology, abundance, and intracellular distribution of mitochondria, which are essential for maintaining cellular homeostasis [51]. In response to pathological stress, mitochondrial dynamics are modulated through fission, fusion, mitophagy, and transport mechanisms, which contribute to minimizing cellular damage by regulating metabolism, energy production, and ion homeostasis [52]. However, when pathological stress exceeds the capacity for regulating mitochondrial dynamics, it can lead to changes in cellular phenotype related to disease.

Alterations in mitochondrial dynamics during cancer development have been studied [53]. Because changes in mitochondrial shape represent changes in metabolic capacity, alterations in mitochondrial dynamics have been suggested as an underlying mechanism of oncogene-mediated metabolic reprogramming [53]. In particular, the roles of the MAPK and PI3K-Akt pathways in regulating mitochondrial dynamics have been reported. Upregulated oncogenic MAPK signaling (RAS-RAF-ERK) is associated with mitochondrial fission. The PI3K-Akt pathway activates mitochondrial fission and mitophagy in tumors.

Although both MAPK and MYC signaling pathways exhibit oncogenic potential, they exert distinct effects on mitochondrial dynamics. While MAPK signaling promotes mitochondrial fission, overexpression of oncogenic MYC enhances mitochondrial biogenesis and fusion [53]. These differences are thought to arise from their distinct regulatory mechanisms, as MAPK rapidly modulates protein activity through phosphorylation, whereas MYC regulates gene expression. Consequently, their roles in regulating mitochondrial dynamics may reflect their unique contributions to metabolic plasticity during carcinogenesis.

Post-transcriptional regulatory machinery in cancer cells can influence the control of mitochondrial dynamics. Heterogeneous nuclear ribonucleoproteins H and F (hnRNP H/F) are known to alter RNA splicing in cancer cells [54]. A recent study reported that hnRNPH1 mediates retrograde signaling in response to mitochondrial damage. Following mitochondrial stress, hnRNPH1 accumulates in the nucleus, where it enhances the transcription of dynamin-related protein 1 (DRP1)—a key mitochondrial fission protein—and promotes its translocation to the mitochondria [55].

Alterations in mitochondrial dynamics are associated with the regulation of epithelial–mesenchymal transition (EMT). In a previous study, pharmacological inhibition of mitochondrial fission suppressed EMT in ovarian cancer in a mouse xenograft model [56]. Another study reported that co-treatment with carbonyl cyanide-4(trifluoromethoxy)phenylhydrazone, an uncoupling reagent of the mitochondrial ETC, and oligomycin, a mitochondrial ATP synthase inhibitor, induced EMT in oral squamous cancer cells [57].

Cancer cells also exploit mitochondrial dynamics as an adaptive survival mechanism to evade cell death. Thomas et al. [58] reported that chemotherapy activates the SIRT1/PGC1α pathway, inducing the expression of genes involved in mitochondrial biogenesis and oxidative phosphorylation. Zhao et al. [59] reported that upregulation of Drp1 is associated with breast cancer metastasis. Cancer cells exchange mitochondria with other cancer cells and key components of the tumor microenvironment, including immune cells, neurons, and endothelial cells. Through this mitochondrial exchange mechanism, cancer cells gain multiple survival advantages by suppressing immune responses, evading immune surveillance, and enhancing metabolic adaptability [60]. Saha et al. [61] reported that intercellular nanotubes mediate mitochondrial transfer between cancer and CD8^+^ T cells, leading to the inactivation of immune cells. A recent study suggested that neuron-to-cancer mitochondrial transfer enhances the oxidative phosphorylation capacity and stemness of cancer cells [62].

### 2.4. Mitochondrial Retrograde Signaling in Cancer

The nucleus regulates gene expression and mitochondrial activity through anterograde signaling pathways. In contrast, mitochondria can initiate retrograde responses that activate nuclear gene expression [63]. The mitochondrial retrograde response compensates for dysfunction caused by various stressors [64]. TCA cycle intermediates, cytochrome C, ROS, mtDNA, mitochondrial peptides, and calcium mediate these signaling pathways [65]. Mitochondrial retrograde signaling affects the inflammatory response, epigenetic regulation, and metabolic reprogramming in tumors [66].

Production of biosynthetic intermediates through truncation of the TCA cycle is a key element of the retrograde response [64]. Although anaerobic glycolysis has long been considered a hallmark of cancer metabolism, recent studies suggest that cancer cells also rely on the ETC as a major source of ATP production [67]. Alterations in TCA cycle metabolites in cancer cells are largely attributed to disruptions in cycle integrity, particularly at the levels of isocitrate dehydrogenase (IDH), succinate dehydrogenase (SDH), and fumarate hydratase (FH) [67].

Especially, defects in SDH leads to increased accumulation and extracellular secretion of succinate [68]. Succinate accumulation suppresses α-KG-dependent enzymes, including 2-oxoglutarate-dependent dioxygenase, prolyl hydroxylase, and ten-eleven translocation proteins, which are engaged in protein hydroxylation, histone and DNA demethylation, collagen biosynthesis, and energy metabolism [68]. Loss-of-function mutations in the SDHB subunit of SDH in tumors induce DNA hypermethylation and promote epithelial–mesenchymal transition (EMT) through HIF-2α activation [69]. Similarly, treatment with dimethyl succinate—a plasma membrane-permeable structural analog of succinate—was reported to enhance cancer cell stemness and induce EMT in mammary epithelial cells by decreasing 5-hydroxymethylcytosine (5hmC) levels in chromatin [70].

Other TCA cycle intermediates have also been investigated in cancer progression [67]. A recent study showed that prolyl 4-hydroxylase 1 (P4HA1)-induced perturbations in α-ketoglutarate (α-KG) and succinate metabolism contribute to antitumor immunity by regulating the activation and exhaustion of CD8^+^ T cells [71]. Citrate accumulation has been associated with the suppression of cancer proliferation through inhibition of the IGF-1R/AKT pathway [72], while the accumulation of succinyl-CoA, succinate, and fumarate has been linked to epigenetic regulation in cancer [67]. In addition, tumors utilize lactate and glutamine as alternative fuels in the TCA cycle, and both have been implicated in malignant transformation [73,74].

Excessive mitochondrial ROS production induces protein misfolding in mitochondria and activates UPR^mt^. UPR^mt^ is a transcriptional regulatory mechanism activated by the accumulation of unfolded proteins within mitochondria, which in turn induces the expression of stress-responsive transcription factors [75]. ATF5, ATF4/CHOP, estrogen receptor alpha (ERα), Heat Shock Factor 1 (HSF1), and SIRT3 are representative mediators of UPR^mt^ [76]. Interestingly, some studies have suggested the role of mtDNA in the regulation of UPR^mt^ [77,78]. Activation of UPR^mt^ is associated with cancer cell survival and resistant to apoptosis [76,79]. Kenny et al. [80] reported that mitohormesis mediates the metastasis of breast cancer by activating UPR^mt^. A recent study has reported that ATF5 is highly expressed in the lung, breast, bladder, and ovarian cancers compared with that in normal tissues [81]. In addition, HSP60, a known target of ATF5, promotes bioenergetic functions in invasive prostate cancer by upregulating β-catenin signaling [82]. SOD2 is a mitochondrial antioxidant gene that can be upregulated by the UPR^mt^, and it exhibits both tumor-suppressive and tumor-promoting functions through scavenging superoxide and regulating hydrogen peroxide levels [83]. The dichotomous role of SOD2 in cancer may be attributed to the diverse roles of ROS in oncogenic pathways, which vary according to their source and the stage of tumor development.

### 2.5. Mitochondrial Stress-Induced ISR in Cancer

ISR is a pivotal signaling network that responds to proteostasis defects by modulating the protein synthesis rate through phosphorylation of the translation initiation factor [84]. Phosphorylation of eukaryotic translation initiation factor 2A (eIF2α) is a major event of the ISR pathway which globally suppresses protein synthesis and enhances the translation of ATF4 mRNA. ATF4 protein sequentially initiates adaptive gene expression [85]. Defects in mitochondrial quality control induce ISR and lead to functional impairment in various organs, including pancreatic β-cells [86], liver [87], heart [88], and brain [89].

Heme-regulated inhibitor (HRI), also known as the heme-regulated eIF2α kinase, is activated by heme deficiency and reduces the formation of the translational initiation complex composed of eIF2, GTP, and Met-tRNAi [90]. Recent studies have highlighted the anticarcinogenic effects of HRI activators, and the HRI–ISR pathway is being explored as a potential molecular target for cancer therapy [85,90]. Pharmacological activation of HRI promotes apoptosis in hematologic malignancies [78]. Also, HRI expression is increased in tumors [91,92], and degradation of HRI by BRIC6-mediated ubiquitination promotes the survival of cancer cells [91].

A recent study reported that the OMA1–DELE1 pathway links mitochondrial damage with HRI-mediated ISR activation [84,92]. Mechanistic studies suggest that OMA1, a metalloprotease activated by mitochondrial damage, cleaves DELE1, which then accumulates in the cytosol, where it interacts with HRI to promote eIF2α phosphorylation and suppress translation initiation [84]. Further studies are needed to determine whether OMA1 and DELE1 could serve as novel therapeutic targets in cancer through regulation of the ISR pathway.

**Figure 1:**
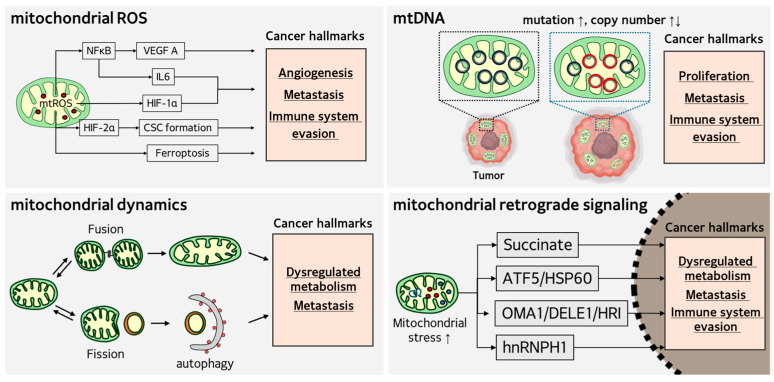
Role of mitochondria in cancer development and progression. Mitochondrial ROS overproduction, mtDNA damage, dysregulation of mitochondrial dynamics, and mitochondrial retrograde signaling are associated with cancer hallmarks. These events represent putative mechanisms that contribute to malignant cell transformation and cancer cell survival.

Taken together, Figure 1 summarizes the role of mitochondria in multiple cancer hallmarks involved in cancer development and progression.

## 3. Dicarbonyl Stress and Mitochondrial Dysfunction

### 3.1. Production and Detoxification of Reactive Dicarbonyls

Elevated levels of reactive dicarbonyls (e.g., glyoxal, MG, and 3-deoxyglucosone) are associated with age-related diseases, including diabetes and cancer, which are characterized by a loss of hormetic mechanisms [93]. In humans, dicarbonyls are typically detected at 50–150 nM in the plasma and 1–4 μM in the cells [94,95]. Endogenous sources of reactive dicarbonyls include sugars, glycated proteins, and lipids [95]. Dicarbonyls are endogenously produced by autoxidation or Maillard reaction of sugars, degradation of glycated proteins, and non-enzymatic peroxidation of polyunsaturated fatty acids [96]. Dicarbonyls are primarily detoxified by glyoxalase and aldehyde dehydrogenase [97].

Mitochondrial fitness, defined as the biological efficiency and functional adequacy of mitochondria [98], is closely associated with the production and removal of reactive dicarbonyls, which in turn influence mitochondrial fitness [93,99]. MG is a byproduct of glycolysis and is removed by a glyoxalase-mediated detoxification mechanism [100]. Oxidative stress is a major driving force that increases MG levels in cells because the production and detoxification of MG are highly dependent on the redox status of glutathione [101]. Mitochondria regulate redox metabolism by generating ROS and storing or utilizing free iron [102], and the impairment of these mechanisms leads to increased oxidative stress.

The role of mitochondria in the regulation of dicarbonyl levels has been previously investigated. Glyoxalase-2 and aldehyde dehydrogenase are expressed in the mitochondria and contribute to the detoxification of dicarbonyls [97]. A novel role of mitochondrial proteins, including mitochondrial ES1 [103] and GATD3A [104], in regulating intracellular MG levels has been reported. In addition, a recent study has suggested that skeletal muscle uncoupling protein-1, which is expressed in the mitochondrial inner membrane, regulates muscular and systemic MG-modified protein levels [105].

### 3.2. Effects of Dicarbonyl Stress on Mitochondrial Function

Dicarbonyl stress is defined as an abnormal accumulation of α-oxoaldehyde metabolites leading to increased modification of protein and DNA [95]. Glycation of mitochondrial proteins has been suggested as a mechanism underlying chronic diseases [106,107]. The accumulation of MG-H1 in the mitochondria has been observed in neurons derived from glyoxalase-1 knockout human induced pluripotent stem cells (Glo1-KO hiPSCs) [108], and increased content of reactive dicarbonyls in the mitochondria has been reported in a mouse model of diabetes [109] and aged mice [110]. Glo1 overexpression reduces mitochondrial MG and ROS in the mouse brain [110]. Glo1-KO induces a decrease in mitochondrial membrane potential and bioenergetic functions in hiPSC-derived neurons [108]. In addition, the renal and cardioprotective effects of MitoGamide, a mitochondria-targeted dicarbonyl scavenger, have been reported in a diabetic mouse [111,112].

Glycation of mitochondrial proteins is associated with increased ROS and energy production [113,114]. Increased ROS production and decreased respiration were observed in MG-treated mitochondria [115]. MG induces mitochondrial ROS production and decreases mitochondrial bioenergetics in the brain endothelial cells [116] and retinal pigment epithelium [117]. Glyoxal decreases mitochondrial membrane potential and activates the MAPK pathway involving ERK, c-Jun N-terminal kinase (JNK), and p38 [118]. In a previous study, oral administration of MG suppressed mitochondrial bioenergetics in the rat brain [119] and exercise-induced changes in mitochondrial function in mouse skeletal muscles [120].

**Figure 2:**
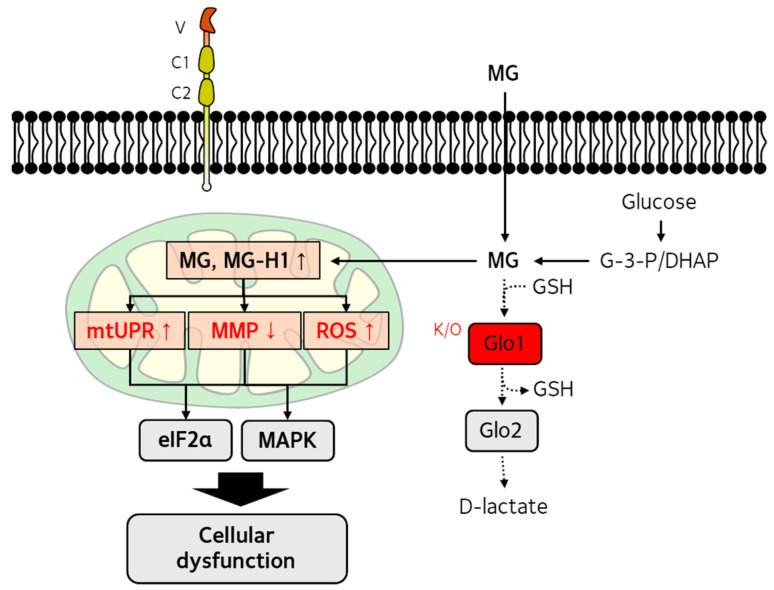
Suggested mechanism of methylglyoxal (MG)-induced mitochondrial damage. The underlying mechanisms of MG-induced mitochondrial dysfunction have been investigated using MG-treated cells or glyoxalase (Glo) knock-out models. These studies revealed activation of the mitochondrial unfolded protein response (mtUPR), decreased mitochondrial membrane potential (MMP), and increased production of mitochondrial reactive oxygen species (ROS). MG-induced mitochondrial damage can further activate eIF2α and MAPK pathways, leading to cellular dysfunction.

The relationship between dicarbonyl stress and UPR has been investigated [121,122]. Accumulation of misfolded MG-modified proteins has been associated with the activation of HSF1 and X-box Binding Protein 1 (XBP1)-mediated UPR [121]. Increased phosphorylation of eIF2α and protein expression of CHOP were reported in Glo-1-knockdown human aortal endothelial cells [123]. Moreover, MG treatment increased the phosphorylation of eIF2α and protein expression of ATF4/CHOP in the retinal pigment epithelium [117]. Figure 2 illustrates the molecular mechanism of MG-induced mitochondrial damage.

## 4. AGE-RAGE Axis and Mitochondrial Dysfunction

Receptor of AGEs (RAGE) is a multi-ligand receptor expressed in various organs. The AGE–RAGE axis triggers a series of downstream signaling pathways, including the NADPH oxidase, NF-κB, MAPK, and JAK/STAT pathways [124]. Activation of the AGE–RAGE axis promotes ROS-mediated signaling, leading to inflammatory response [125]. Several studies have reported an association between the AGE–RAGE axis and mitochondrial dysfunction in AGE-treated cells and animals.

As shown in Table 1, the AGE diet induced mitochondrial dysfunction in various organs, including the brain, heart, kidney, and skeletal muscle in animals. Notably, the AGE diet decreased mitochondrial bioenergetics [126,127,128] and dynamic dysregulation [129]. In these studies, mitochondrial damage was associated with oxidative stress [129], ER stress [130], JNK activation [130], and inflammatory response [127]. RAGE knockout reversed the effects of high-fat diet on mitochondrial dynamic dysregulation [129] and mitochondrial ETC enzyme activity (Complex II + III, IV) [127].

**Figure 3:**
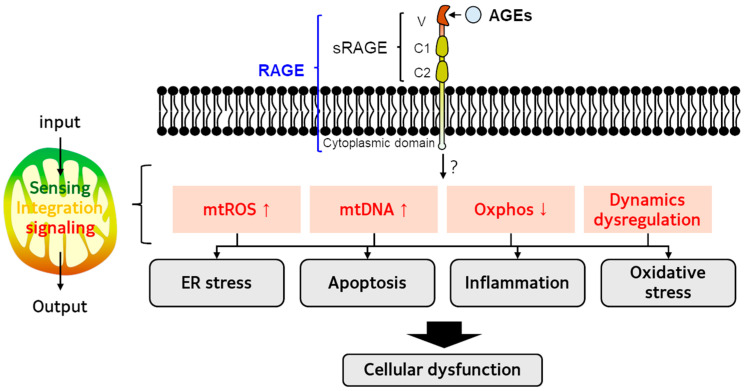
Crosstalk between RAGE–AGEs axis and mitochondrial network. The underlying mechanisms of AGEs-induced mitochondrial dysfunction have been investigated. Mitochondria sense AGEs as intracellular stress signals and contribute to cellular dysfunction by altering the mitochondrial network. Activation of the RAGE–AGEs axis results in mitochondrial reactive oxygen species (mtROS) overproduction, mitochondrial DNA (mtDNA) damage, impaired oxidative phosphorylation (Oxphos), and dysregulated mitochondrial dynamics, which collectively increase endoplasmic reticulum (ER) stress, apoptosis, inflammation, and oxidative stress, ultimately leading to cellular dysfunction.

In vitro studies on AGE-induced mitochondrial dysfunction are described in Table 2. AGE treatment impaired mitochondrial bioenergetics and dynamics regulation. Also, oxidative stress [128], ER stress [130], apoptosis [131], and extracellular matrix remodeling [132], as well as mitochondrial damage, were observed in AGE-exposed cells. Figure 3 suggests the mechanisms of crosstalk between the AGE-RAGE axis and mitochondrial network.

## 5. AGEs and Cancer

### 5.1. Epidemiology Studies

Diabetes has been associated with an increased cancer risk [133,134], and increased AGE levels are putative mechanisms that explain the biological plausibility of the association between hyperglycemia and increased cancer risk. Several epidemiological studies have reported an association between the dietary intake of AGEs and an increased risk of gallbladder [135], pancreatic [136], and breast cancers [137,138], and another multinational cohort study reported no association between dietary AGEs and overall cancer risk [139]. In addition, increased serum AGEs and mRNA expression of RAGE in cancerous tissue have been reported in patients with gastric cancer [140], and serum AGE concentration was correlated with metastasis of breast cancer in a cohort study [141]. Peterson and Ligibel recently reviewed the relationship between dietary or serum AGEs and epidemiological outcomes in breast cancer [142]. The study reported an association between dietary AGEs and an increased incidence of breast cancer but also acknowledged the limitations of existing research arising from non-uniform study designs [142].

### 5.2. Animal Studies

The effects of exogenous and endogenous AGEs on tumor growth have been studied in normal animals and tumor xenograft models, as shown in Table 3. The effect of high-AGE diet on mammary gland development was investigated in juvenile mice, and atypical hyperplasia in mature mammary glands was observed after the intake of a high-AGE diet [143]. In addition, dietary early glycation products (EGPs) promoted tumor growth and progression in prostate [144] and pancreatic cancer models [145]. In a streptozotocin-induced type 1 diabetes model, enhanced lung metastasis of chondrosarcomas was observed, along with increased blood CML levels [146]. In contrast, one study reported the inhibitory effect of dietary AGEs on the growth of a subcutaneously inoculated non-small cell lung cancer (NSCLC) cell line [147]. The authors stated that some AGE structures might be associated with tumor growth-inhibiting effects [147].

Other studies investigated the role of the RAGE pathway in cancer development and progression, as shown in Table 4. RAGE knockout suppressed cancer growth in the liver [148] and pancreatic cancer [149] and alleviated the immunosuppressive microenvironment in pancreatic cancer [149]. RAGE and AGE aptamers inhibited melanoma by decreasing angiogenesis and immune cell infiltration in tumors [150,151]. In addition, the RAGE aptamer suppressed tumor growth by inhibiting oxidative stress, cell cycle, and liver metastasis [150]. Short hairpin RNA (shRNA) against RAGE suppressed tumor growth and increased the mRNA levels of the death receptors DR4 and DR5 [152]. In a pancreatic cancer model, AGE antibodies suppressed tumor growth and decreased the number of senescent cells in tumors [153].

### 5.3. In Vitro Studies

The tumor-promoting effect of AGEs, including proliferation, migration, and invasion of cancer cells, has been reported in in vitro studies, as shown in Table 5. Increased oxidative stress [150] and activation of the NF-κB pathway [145,146] have been suggested as molecular mechanisms of AGE-induced tumor-promoting effects. AGEs induce phenotypic changes in tumor and immune cells. AGE treatment induces cancer stemness, EMT of tumor cells [146], and polarization of macrophages [144]. The involvement of the AGE–RAGE axis in cancer cell migration and invasion has been demonstrated. Activation of this axis mediates the migration of lung [157], oral [158], and breast cancer cells [159]. Moreover, downregulation of RAGE expression reduces the invasive capacity of colon cancer cells [160]. AGEs increased tube formation in human umbilical vein endothelial cells (HUVECs) [150,151] and induced mRNA expression of vascular endothelial growth factor (VEGF) [150]. In a 3D culture model, AGE treatment increased spheroid size of cells in chondrosarcoma [146] and prostate cancer [144].

### 5.4. AGEs and Mitohormesis Dysregulation in Cancer

Finally, to elucidate the association between AGE-induced mitochondrial dysfunction and cancer development, we integrated and reanalyzed mechanistic evidence related to the hallmarks of cancer [6,161]. Procarcinogenic or anticarcinogenic functions of signaling molecules involved in cancer development have been suggested by Goodson et al. [161].

Table 6 summarizes the mechanisms underlying AGE-related carcinogenesis, molecular functions, changes in the expression or activity, and carcinogenic effects. A total of 11 signaling molecules were identified from the previously mentioned study on AGE-induced carcinogenesis. Tumor-promoting inflammation through activation of the NF-κB pathway was reported in two independent studies. However, evidence suggests opposing mechanisms for angiogenesis, immune system evasion, tumor microenvironment, and tumor-promoting inflammation, as shown in Table 6.

As outlined in Section 3 and Section 4, increased dicarbonyl stress and the AGE–RAGE axis place excessive stress on the mitochondrial system, which in turn triggers adaptive cellular responses. These findings suggest that AGEs induce alterations in mitohormesis, potentially contributing to cancer development. Accordingly, we examined mitochondrial alterations linked to dicarbonyl stress and the AGE–RAGE axis in the context of the hallmarks of cancer, as shown in Table 7. Mitochondrial ROS, dynamic regulation, and mtDNA copy number were identified as key components of mitohormesis induced by AGEs. The signaling pathways associated with cancer hallmarks include immune system evasion [28,162], sustained proliferative signaling [24], dysregulated metabolism [85], and invasion and metastasis [42,56] through the activation of the NF-κB, Akt, UPR, and EMT pathways.

**Figure 4:**
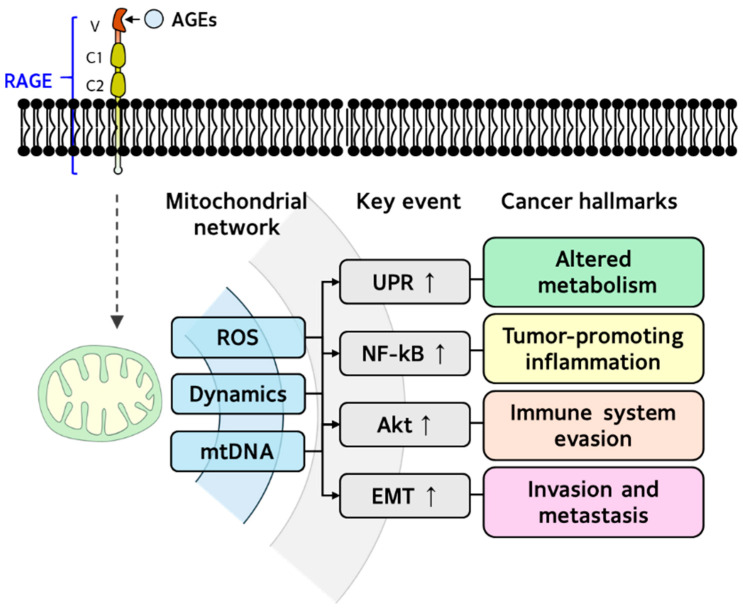
Putative mechanism of cancer hallmark associated with communication between AGEs–RAGE axis and mitochondrial network. The mechanisms of AGEs-induced cancer development and progression are linked to alterations in the mitochondrial network and to cancer hallmarks, including UPR-mediated dysregulated metabolism, NF-κB-driven tumor-promoting inflammation, Akt-mediated immune evasion, and EMT-mediated invasion and metastasis.

As shown in Figure 4, AGE-induced mitochondrial ROS production and dynamic dysregulations are highly associated with the mechanisms of NF-κB-mediated tumor-promoting inflammation and EMT-mediated invasion and metastasis, respectively. These results suggest that elevated reactive dicarbonyls or AGEs function as stressors, disrupting cellular bioenergetic and redox balance, thereby activating mitohormesis and promoting malignant transformation and cancer cell survival.

## 6. Conclusions

To integrate the mechanistic evidence of AGEs-induced cancer, we reviewed the recent studies related to the role of mitochondria in AGEs-induced carcinogenesis. Mitochondria are involved in the formation of AGEs through regulating reactive dicarbonyl levels, and mitochondrial damage caused by AGEs can act as a stressor that contributes to malignant cell transformation. Although the mechanisms by which AGEs induce mitochondrial damage remain unclear, it appears to involve a combination of increased oxidative stress and bioenergetic perturbation in mitochondria. Future in-depth studies focusing on the mitochondrial information processing system framework may allow for a more systematic understanding of key pathway associated with AGEs-induced carcinogenesis. This, in turn, could contribute to the development of strategies for the treatment and prevention of various chronic diseases induced by AGEs.

## Figures and Tables

**Table 1:** In vivo study on crosstalk between AGE–RAGE axis and mitochondrial dysfunction.

Species (Sex)	Target Organ	Treatment	Adverse Outcome		Reference
			Mitochondria	Cell/Organ	
C57BL/6 mice (M, F)	Brain	* AGE diet (1000 mg/kg, 17 months)	** Oxphos capacity ↓ ATP production ↓	Cognitive impairment	[126]
C57BL/6N mice (M)	Kidney	* AGEs diet (800 mg/kg, p.o., 3 weeks)	-	ER stress ATF4/CHOP, GRP78 ↑ p-JNK/JNK ↑	[130]
RAGE-deficient C57BL/6J mice (M)	Skeletal muscle	High-fat diet (4 months)	* Oxphos capacity ↓	Inflammatory response (IL-1β)	[127]
RAGE-deficient C57BL/6J mice (M)	Heart	High-fat diet (4 months)	Mitochondria count ↓ Morphological change	Oxidative stress	[129]
SD rats (M)	Kidney	AGEs (20 mg/kg, i.p., 16 weeks)	* Oxphos capacity ↓ Mitochondrial NADH ↓	-	[128]

* Methylglyoxal-derived AGEs. ** Oxphos: oxidative phosphorylation.

**Table 2:** In vitro study on AGE-induced mitochondrial dysfunction.

Cell Type	Origin	AGE Treatment	Adverse Outcome		Reference
			Mitochondria	Cell	
Kidney proximal epithelial cell line (HK-2)	Human	200 μg/mL, 24 h (MG-derived AGE)	ATP production ↓ MMP ↓	ER stress ATF4/CHOP, GRP78 ↑ p-JNK/JNK ↑	[130]
Osteoblastic cell line (MC3T3-E1)	Mouse	* 400 μg/mL, 24 h	ROS production ↑ ATP production ↓ MMP ↓, fission ↑	Apoptosis	[131]
RAGE-overexpressed primary mesangial cells	Rat	100 μg/mL, 48 h (Glucose-derived AGE)	** Oxphos capacity ↓ Mitochondrial permeability transition ↑	Oxidative stress	[128]
Primary rat fibroblast	Rat	500 μg/mL, 0.5 h (Glycolaldehyde-derived AGE)	mtDNA count ↑ ROS production ↑ ATP production ↓	Extracellular matrix remodeling	[132]

* Composition of AGE was not reported. ** Oxphos: oxidative phosphorylation.

**Table 3:** Effect of endogenous and exogenous AGEs on cancer development and progression.

Cancer Type	Animals (Sex)	Treatment (Dosage, Route, Duration)	Adverse Outcome	Reference
Breast cancer	FVB/n mice (F)	15–25 g AGE diet/week, p.o., 4–25 weeks	▫ Atypical hyperplasia in mammary gland	[143]
Chondrosarcoma	NOD/SCID mice (M)	40 mg streptozotocin/kg body weight/day, 5 days	▫ Increased blood CML level ▫ Enhanced tumor metastasis in chondrosarcoma without affecting tumor growth	[146]
Lung cancer	NMRI nu/nu mice (F)	6 g AGE diet/day, p.o., 14 days	▫ Decreased tumor weight and volume	[147]
Prostate cancer	C57BL/6 mice (M)	600 mg EGPs/kg body weight/day, p.o., 4 weeks	▫ Promoted growth of transplanted prostate cancer and increased circulating tumor-associated M2 macrophages	[144]
Pancreatic cancer	KC mice (N/A)	30 μg AGE/day, i.p., 6 weeks	▫ Accelerated progression to invasive pancreatic cancer	[145]

**Table 4:** Animal studies on RAGE-mediated cancer development.

Cancer Type	Animals (Sex)	Treatment (Dosage, Route, Duration)	Adverse Outcome	Reference
Breast cancer	C57BL6 mice (N/A)	RAGE knockout	▫ Decreased tumor growth ▫ Decreased angiogenesis and leukocyte infiltration	[154]
	C57B/6 mice (N/A)	RAGE knockout	▫ Decreased tumor volume and weight	[155]
Liver cancer	Mdr2^−/−^ C57Bl/6 mice (M)	RAGE knockout	▫ Decreased size and number of hepatocellular carcinoma cells	[148]
Lung cancer	BALB/c nude mice (M)	Inoculating RAGE-overexpressed A549	▫ Increased tumor volume ▫ Increased tumor-associated macrophages	[156]
Prostate cancer	Nude mice (M)	shRAGE (100 μg, 5 times/week, i.p., 6 weeks)	▫ Decreased tumor volume ▫ Decreased mRNA expression of RAGE and HMGB1 ▫ Increased mRNA expression of DR4 and DR5	[152]
Pancreatic cancer	Humanized CD34^+^ NSG mice (F)	AGE antibody (10 or 20 mg/kg BIW × 1 followed by 5 or 10 mg/kg BIW × 2)	▫ Suppressed tumor growth ▫ Increased complete remission rate ▫ Decreased senescent cells in the tumor microenvironment	[153]
	Pdx1-Cre:Kras^G12D/+^ C57BL/6 mice (N/A)	RAGE knockout	▫ Delayed carcinogenesis ▫ Less suppressive milieu (decrease in CCL22:CXCL10 and IL-6)	[149]
Skin cancer	Nude mice (F)	RAGE aptamer (38.4 pmol/day/g body weight, i.p., 42 days)	▫ Inhibited tumor growth ▫ Reduced 8-OHdG, AGEs, RAGE, proliferating nuclear antigen, cyclin D1, VEGF, MCP-1, CD31, and Mac-3 in tumors ▫ Suppressed liver metastasis	[150]
	Athymic nude mice (F)	AGE aptamer (0.136 μg/day, i.p., 43 days)	▫ Inhibited tumor growth ▫ Decreased expression levels of proliferating nuclear antigen, CD31, Mac-3, and VEGF ▫ Decreased tumor-associated vessels	[151]

**Table 5:** In vitro mechanistic studies of tumor-promoting effects of AGEs.

Cell Type	Origin	Treatment	Adverse Outcome	Reference
Chondrosarcoma cell line (JJ012, SW1353)	Human	25–100 μM CML, 24–72 h	▫ Increased protein expression levels of RAGE and phosphorylated NFκB-p65 ▫ Decreased phosphorylation of Akt and GSK-3 ▫ Enhanced tumor-sphere formation and expression of cancer stem cell marker ▫ Enhanced migration and invasion and epithelial–mesenchymal transition	[146]
Human umbilical vein endothelial cells (HUVECs)	Human	100 μg/mL AGE-BSA, 24 h	▫ Increased proliferation and tube formation	[151]
		50 μg/mL AGE-BSA, 4 h	▫ Increased mRNA expression of VEGF, MCP-1, and VCAM-1 ▫ Increased superoxide production and cell proliferation ▫ Increased tube formation ▫ Increased human monocyte (THP-1) adhesion	[150]
Melanoma cell line (G361)	Human	1000 μg/mL AGE-BSA, 24 h	▫ Increased proliferation	[151]
		1000 μg/mL AGE-BSA, 24 h	▫ Increased protein expression of CyclinD1/p27 and mRNA expression of VEGF and MCP-1 ▫ Increased superoxide production and cell proliferation	[150]
Prostate cancer cell line (LNCaP) /PMA-differentiated macrophages (d-U937)	Human	2.5 mg/mL early glycation products (EGPs), 48 h	▫ Increased spheroid size and polarization of macrophages (M2) ▫ Decreased iNOS expression	[144]
Pancreatic ductal adenocarcinoma cell line (PANC-1)	Human	50 μg/mL CML, 24 h	▫ Activation of the NF-κB–STAT3–PIM1–NFAT axis ▫ Increased cell proliferation	[145]
Primary mammary fibroblast /Mammary epithelial cell-line (HC11)/mammary gland carcinoma (Met1)	Mouse	50 μg/mL BSA-AGE, 24 h	▫ Promoted epithelial migration and invasion of tumor-derived mammary epithelial cells	[143]

**Table 6:** Key mediators associated with AGE-induced carcinogenesis.

Cancer Hallmarks	Key Mediators	Changes	Effect	Reference
Angiogenesis	CXCL10	Decreased	Anticarcinogenic	[149]
Dysregulated metabolism	ROS	Increased	Procarcinogenic	[150]
Immune system evasion	Akt	Decreased	Anticarcinogenic	[146]
	CXCL10	Decreased	Anticarcinogenic	[149]
Sustained proliferative signaling	Cyclin D1	Increased	Procarcinogenic	[150]
Activating invasion and metastasis	EMT	Increased	Procarcinogenic	[146]
Tumor microenvironment	Oxidative stress	Increased	Procarcinogenic	[150]
	IL-6	Decreased	Anticarcinogenic	[149]
Tumor-promoting inflammation	NFκB	Increased	Procarcinogenic	[145,146]
	IL-6	Decreased	Anticarcinogenic	[149]

**Table 7:** Mitohormesis signaling and response induced by AGEs and their association with carcinogenic effects.

Mitohormesis		Cancer Hallmarks	Effect	Reference
Signaling	Response			
mtROS	NF-κB ↑	Tumor-promoting inflammation	Procarcinogenic	[131,132]
	Akt ↑	Immune system evasion	Procarcinogenic	
	* UPR ↑	Dysregulated metabolism	Procarcinogenic	
Dynamics	EMT ↑	Invasion and metastasis	Procarcinogenic	[131]
* mtDNA	N/A	Invasion and metastasis	Procarcinogenic	[132]

* Refs. [85,42] reported cancer hallmarks associated with mitochondrial stress-induced UPR and mtDNA.

## Data Availability

No new data were created or analyzed in this study. Data sharing is not applicable to this article.

## Abbreviations

3-DG	3-deoxyglucosone
5hmC	5-hydroxymethylcytosine
AGEs	advanced glycation end products
CLL	chronic lymphocytic leukemia
CML	Nε-(carboxylmethyl)-l-lysine
CYTB	cytochrome B
DRP1	dynamin-related protein 1
EGPs	early glycation products
eIF2α	eukaryotic translation initiation factor 2A
EMT	Epithelial–mesenchymal transition
ERK	extracellular signal-regulated kinase
ERα	estrogen receptor alpha
ETC	electron transport chain
FH	fumarate hydratase
Glo1-KO hiPSCs	glyoxalase-1 knockout human induced pluripotent stem cells
HIF-1α	hypoxia-inducible factor 1α
hnRNP	H/F heterogeneous nuclear ribonucleoproteins H and F
HRI	Heme-regulated inhibitor
HSF1	Heat Shock Factor 1
HUVECs	human umbilical vein endothelial cells
IDH	isocitrate dehydrogenase
ISR	integrated stress response
JNK	c-Jun N-terminal kinase
MAPK	mitogen-activated protein kinase
MG	methylglyoxal
MG-H1	Nδ-(5-hydro-5-methyl-4-imidazolon-2-yl)-l-ornithine
mtDNA	mitochondrial DNA
NOX	nicotinamide adenine dinucleotide phosphate oxidase
NSCLC	non-small cell lung cancer
PD-1	programmed cell death protein 1
ROS	reactive oxygen species
SDH	succinate dehydrogenase
SGs	stress granules
shRNA	short hairpin RNA
SIRT3	Sirtuin 3
SOD2	superoxide dismutase 2
TCA	tricarboxylic acid
TIL	tumor-infiltrating lymphocyte
UPRmt	mitochondrial unfolded protein response
VEGF	vascular endothelial growth factor
XBP1	X-box Binding Protein 1
XO	xanthine oxidase
